# High-CPAP Does Not Impede Cardiovascular Changes at Birth in Preterm Sheep

**DOI:** 10.3389/fped.2020.584138

**Published:** 2021-01-22

**Authors:** Tessa Martherus, Kelly J. Crossley, Karyn A. Rodgers, Janneke Dekker, Anja Demel, Alison M. Moxham, Valerie A. Zahra, Graeme R. Polglase, Calum T. Roberts, Arjan B. te Pas, Stuart B. Hooper

**Affiliations:** ^1^Division of Neonatology, Department of Pediatrics, Leiden University Medical Center, Leiden, Netherlands; ^2^The Ritchie Centre, Hudson Institute of Medical Research, Melbourne, VIC, Australia; ^3^Department of Obstetrics and Gynecology, Monash University, Melbourne, VIC, Australia; ^4^Monash Newborn, Monash Medical Centre, Melbourne, VIC, Australia

**Keywords:** preterm, CPAP, pulmonary blood flow, cerebral blood flow, jugular venous pressure, birth

## Abstract

**Objective:** Continuous positive airway pressures (CPAP) used to assist preterm infants at birth are limited to 4–8 cmH_2_O due to concerns that high-CPAP may cause pulmonary overexpansion and adversely affect the cardiovascular system. We investigated the effects of high-CPAP on pulmonary (PBF) and cerebral (CBF) blood flows and jugular vein pressure (JVP) after birth in preterm lambs.

**Methods:** Preterm lambs instrumented with flow probes and catheters were delivered at 133/146 days gestation. Lambs received low-CPAP (LCPAP: 5 cmH_2_O), high-CPAP (HCPAP: 15 cmH_2_O) or dynamic HCPAP (15 decreasing to 8 cmH_2_O at ~2 cmH_2_O/min) for up to 30 min after birth.

**Results:** Mean PBF was lower in the LCPAP [median (Q1–Q3); 202 (48–277) mL/min, *p* = 0.002] compared to HCPAP [315 (221–365) mL/min] and dynamic HCPAP [327 (269–376) mL/min] lambs. CBF was similar in LCPAP [65 (37–78) mL/min], HCPAP [73 (41–106) mL/min], and dynamic HCPAP [66 (52–81) mL/min, *p* = 0.174] lambs. JVP was similar at CPAPs of 5 [8.0 (5.1–12.4) mmHg], 8 [9.4 (5.3–13.4) mmHg], and 15 cmH_2_O [8.6 (6.9–10.5) mmHg, *p* = 0.909]. Heart rate was lower in the LCPAP [134 (101–174) bpm; *p* = 0.028] compared to the HCPAP [173 (139–205)] and dynamic HCPAP [188 (161–207) bpm] groups. Ventilation or additional caffeine was required in 5/6 LCPAP, 1/6 HCPAP, and 5/7 dynamic HCPAP lambs (*p* = 0.082), whereas 3/6 LCPAP, but no HCPAP lambs required intubation (*p* = 0.041), and 1/6 LCPAP, but no HCPAP lambs developed a pneumothorax (*p* = 0.632).

**Conclusion:** High-CPAP did not impede the increase in PBF at birth and supported preterm lambs without affecting CBF and JVP.

## Introduction

Respiratory support for preterm infants at birth has shifted from intubation and mechanical ventilation toward non-invasive strategies (1–4). However, when applied non-invasively, intermittent positive pressure ventilation (iPPV) is unable to ventilate the lung if the larynx is closed, which is known to occur in the fetus and newborn during apnea (5–8). As such, attention has now focused on stimulating and supporting spontaneous breathing at birth using continuous positive airway pressure (CPAP) (9–11). This has highlighted a knowledge gap regarding how CPAP should be applied in the delivery room. Currently, 4–8 cmH_2_O of CPAP is widely adopted, but this has been extrapolated from strategies used in the neonatal intensive care unit as there is little scientific evidence to support this pressure range at birth (12, 13).

To optimize CPAP support for preterm infants at birth, the underlying physiology of the infant needs to be considered as it transitions to newborn life. During pregnancy, the airways are liquid-filled and pulmonary blood flow (PBF) is low and so at birth, the airways must be cleared of liquid to allow the entry of air and PBF must increase to facilitate the onset of pulmonary gas exchange (14–18). Lung aeration triggers a decrease in pulmonary vascular resistance (PVR) and increase in PBF (19, 20), which is critical for the maintenance of cardiac output after birth, as PBF must take over the role of providing preload for the left ventricle following cord clamping (17).

As transpulmonary pressures generated by inspiration drive lung aeration after birth, increasing this pressure gradient could assist spontaneously breathing newborns aerate their lungs (18, 21, 22). This can be achieved by applying CPAP, but the optimal CPAP strategy is unknown. As airway resistance is initially high, due to the high viscosity of airway liquid (23, 24), theoretically, a high-CPAP will help overcome the initial high resistance of the liquid-filled airways. However, as the lungs aerate the resistance reduces and so the required CPAP level may also reduce (18, 22–25). Thus, a CPAP strategy that starts high and then decreases could reflect a changing physiological role for CPAP at birth. Initially CPAP may assist with lung aeration, but as the lung aerates and liquid is replaced by air, the role of CPAP could change to preventing liquid re-entry and alveolar collapse (18, 22–25). However, high airway pressures have been associated with pulmonary overexpansion, pneumothoraxes, reductions in cardiac output and PBF as well as elevated central venous pressures in (preterm) infants (26–28), adults (29–32), and animals (33–36).

Indeed, while high positive end-expiratory pressures (PEEP) enhance lung aeration and oxygenation and can reduce intubations and surfactant requirements in preterm infants (37), they also reduce PBF and increase the risk of pneumothoraxes in intubated and mechanically ventilated animals (38–42). As the application of CPAP during spontaneous breathing and PEEP during mechanical ventilation may have different effects on lung physiology, it is important to understand how high-CPAP levels affect lung physiology during spontaneous breathing. Recently, a retrospective study across two hospitals has compared two different CPAP levels (5–8 cmH_2_O vs. 12–35 cmH_2_O) in preterm infants. While they found no differences in oxygenation levels or heart rates, high-CPAP was found to reduce the use of supplemental oxygen at the expense of a higher pneumothorax rate (43). The latter may result from pulmonary overexpansion, but similar oxygenation levels and heart rates indicate that the cardiovascular system was not compromised. Although high intra-thoracic pressures are known to reduce venous return, increase central venous pressure, reduce cardiac output and reduce PBF, the impact of high-CPAP levels during spontaneous breathing on cardiovascular function is unknown (35, 38, 41, 44, 45).

The aim of this study was to determine the effect of high-CPAP levels, applied immediately after birth, on PBF, cerebral blood flow (CBF) and jugular venous pressure (JVP) and to determine whether a decrease in CPAP after stabilization can avoid these adverse effects. We hypothesized that high-CPAP during transition will reduce PBF and CBF and increase JVP, whereas high-CPAP levels that are reduced as the lung aerates will avoid these adverse effects.

## Materials and Methods

### Ethics Statement

Study procedures were performed in accordance with the National Health and Medical Research Council of Australia guidelines for care and use of experimental animals and were approved by Monash University “MMCA” Ethics committee. All research staff exposed to the sheep were vaccinated against Q fever (Q-Vax CSL, Australia).

### Pre-experimental Surgical Preparation

At 129–130 days gestation, ewes (Border-Leicester) were anesthetized for fetal instrumentation. Anesthesia was induced with sodium thiopental (1 g in 20 mL, Pentothal IV; Jurox, NSW, Australia) and maintained, following endotracheal intubation, with inhaled isoflurane (≈2–5%; Isoflow, Abbott Laboratories, IL, US) in air/oxygen as previously described (46). Following exteriorization of the fetus (Merino X Border-Leicester), ultrasonic flow probes (Transonic Systems, Ithaca, NY, US) were placed around the left carotid artery (size 3) and left pulmonary artery (size 4), whereas polyvinyl catheters (0.86 mm ID, Dural Plastics, Sydney, NSW, Australia; 20 G × 1.16 peripheral venous catheter (1.1 mm × 30 mm), BD Insyte^TM^, Franklin Lakes, NJ, US) were inserted into the left brachial artery and right jugular vein. A silastic tube (0.078 and 0.125 mm ID, Down Corning, US) was placed in the upper trachea, and a sterile, saline-filled intrapleural balloon catheter (2.6 mm ID, Dural Plastics, Sydney, NSW, Australia) was placed in the intrapleural cavity. A transdermal fentanyl patch (75 μg/h; Janssen Cilag, Belgium) was used for post-operative analgesia and antibiotics (Cefazolin; AFT Pharmaceuticals, Auckland, New Zealand) were administered to the ewe (1 g), fetus (100 mg), and amniotic sac (400 mg) on the day of surgery, and daily for 2 days post-operatively.

### Delivery and Support

At 132–133 days gestation (equivalent to ~28–30 weeks gestation age in humans), lambs were delivered by cesarean section under spinal anesthesia, as previously described (47). Ewes were initially sedated with intravenous propofol (1 mg/kg; Claris Lifesciences Limited, Gujarat, India) while spinal anesthesia was induced (lignocaine 2%, 0.1 mL/kg, Pfizer, NY, US), following which sedation continued with intravenous midazolam (1.0 mg/kg/h at 20–25 mL/h, Alphapharm, NSW, Australia); local anesthetic (xylocaine 10%, AstraZeneca, Cambridge, UK) was applied to the incision site.

Just prior to full delivery, a transcutaneous pulse oximeter (Radical 7, Masimo, CA, US) was attached to the right forelimb of the lamb to measure peripheral arterial oxygen saturation (SpO_2_) and a Near Infrared Spectroscopy (NIRS) optode (Casmed Foresight, CAS Medical Systems Inc., Branford, CT, US) was placed over the left frontal cortex. Blood gas measurements were corrected to core body temperature measured using a rectal temperature probe (ADInstruments, NSW, Australia). Catheters and probes were connected to a PowerLab (ADInstruments, NSW, Australia) to enable continuous recording of physiological data throughout the study.

The umbilical cord was immediately clamped before lambs were moved to a warmed bed (CosyCot, Fisher and Paykel, Auckland, New Zealand) and were given naloxone (0.01 mg/kg, Juno Therapeutics, WA, US) and anexate (0.1 mg/mL, Flumazenil, Apotex, Toronto, Canada) to counteract any maternally administered fentanyl and midazolam that had crossed the placenta before cord clamping. Also, caffeine (20 mg/kg, Sigma-Aldrich, St. Louis, MO, US) and doxapram (5 mg/kg, Bayer, Leverkusen, Germany) were given to stimulate spontaneous breathing immediately after birth. CPAP was provided by a ventilator in CPAP mode (Babylog 8000+, Drager, GA Lübeck, Germany) using warmed and humidified gases (MR850 Heated Humidifier, Fisher & Paykel, Auckland, New Zealand) and applied using bi-nasal prongs especially designed for preterm sheep. Respiratory support was commenced with CPAP and a fraction of inspired oxygen (FiO_2_) of 0.3. If the lamb showed visible gastric distension, a tube was placed briefly in the esophagus to remove air. The tube was removed after the gastric distension diminished.

If lambs became apneic (defined as an absence of breathing for 4–5 s) or had a decreasing cerebral or peripheral oxygenation, tactile stimulation (rubbing fingers along the lambs spine, limbs, and body) was applied, FiO_2_ was stepwise increased (0.3–0.5–1.0) to target a SpO_2_ of 90–95% and if this failed rescue interventions were given [additional caffeine bolus (20 mg/kg) and/or iPPV (PEEP 8, PIP 35 cmH_2_O 1:1 inflation: deflation time)].

### CPAP Intervention

**Figure 1:**
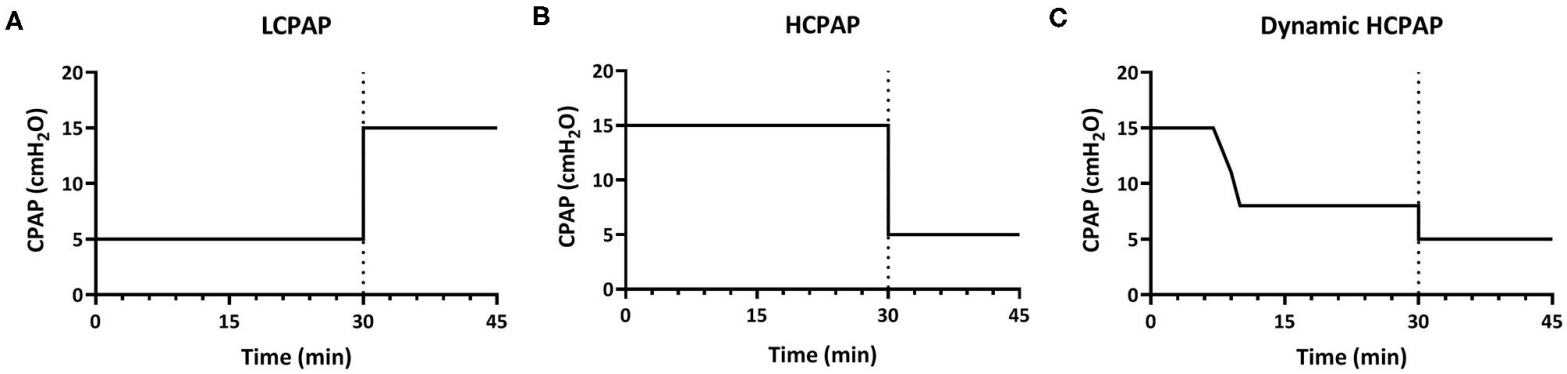
Experimental design. In part 1, lambs were divided into three groups and for 30 min received either low-CPAP [5 cmH_2_O; LCPAP **(A)**], high-CPAP [15 cmH_2_O; HCPAP **(B)**], or high-CPAP that was gradually reduced after reaching set physiological parameters [decreasing from 15 to 8 cmH_2_O; dynamic HCPAP **(C)**]. After 30 min (indicated by dotted line), CPAP was either increased (to 15 cmH_2_O) in the LCPAP group or reduced (to 5 cmH_2_O) in both the HCPAP and dynamic HCPAP groups for a further 15 min (part II).

Lambs received various CPAP levels to (I) compare CPAP-strategies and (II) to explore the effect of changes in CPAP level. The experimental design is detailed below and presented in Figure 1.

#### Part I: Comparing CPAP-strategies (0–30 min)

Lambs were randomly allocated to a low-CPAP (control group: LCPAP, 5 cmH_2_O) or two high-CPAP groups that used different strategies (intervention groups). A CPAP of 5 cmH_2_O was used as the control group as it is widely used in clinical practice and it is well-established that an end-expiratory pressure is required to help aerate the lung of preterm neonates (12, 13). Lambs allocated to the intervention groups received high-CPAP (HCPAP, 15 cmH_2_O) or an initially high-CPAP that was decreased (dynamic HCPAP, 15–8 cmH_2_O). In the dynamic HCPAP group, CPAP was reduced when PBF was >300 mL/min, SaO_2_ >90%, FiO_2_ ≤ 0.7 and the lamb was spontaneously breathing. At this time, CPAP was decreased in a stepwise fashion at a rate of ~2 cmH_2_O per minute to 8 cmH_2_O, which is the upper limit of the recommended CPAP range in the ANZCOR guidelines (48).

#### Part II: Exploring the Effect of CPAP Changes (30–45 min)

In lambs that completed the first 30 min of the experiment, CPAP levels were either increased or decreased and the study continued for a further 15 min to explore the effect of CPAP changes on lung physiology after aeration. In the LCPAP group, CPAP was immediately increased from 5 to 15 cmH_2_O, whereas in the HCPAP group, CPAP was immediately decreased from 15 to 5 cmH_2_O and in the dynamic HCPAP group, CPAP was immediately decreased from 8 to 5 cmH_2_O.

Lambs received a lethal dose of sodium pentobarbitone (100 mg/kg intravenously, Virbac Pty Ltd., Peakhurst, Australia) after completing study duration (30 + 15 min) or earlier due to reaching an ethical endpoint, where it was assessed that intubation was considered necessary. Intubation was deemed necessary if lambs were hypoxic and did not initiate breathing, despite the use of tactile stimulation, 100% oxygen and the rescue interventions detailed above.

### Outcome Measures

#### Physiological Data Measurement and Collection

PBF and CBF were measured using a flow meter (Transonics systems; Ithaca, NY, US), whereas brachial arterial (BAP), intrapleural, upper tracheal (UTP), and jugular venous pressures (JVP) were measured with pressure transducers (BP Transducer/Cable kit, ADInstruments, New South Wales Australia) and FiO_2_ was measured with a MX300-I Oxygen sensor (Teledyne Analytical instruments, CA, US). Physiological data was recorded continuously using LabChart 8 (ADInstruments, New South Wales Australia) and arterial blood gas status was measured every 3 min (ABL90 flex and ABL800 analyzer, Radiometer Medical ApS, Brønshøj, Denmark).

For physiological data, a mean was calculated over a 10 s epoch every 3 min, heart rate was derived from PBF and alveolar to arterial differences in PO_2_ (AaDO_2_) was calculated as [(713^*^FiO_2_) -(PaCO_2_/0.8) -PaO_2_]. To assess CBF stability, a mean CBF was calculated over each 15 s epochs and the CBF variability was calculated by measuring the coefficient of variation of mean CBF values throughout the experiment; this was calculated as [standard deviation (CBF)/average (CBF)^*^100]. Breathing rate (60/duration period^*^number of breaths) and inter-breath variability [standard deviation (duration breath)/average (duration breath)^*^100] were calculated in 30 s epochs. Expirations that involved distinct increases in intrapleural pressure above baseline were considered to be active expirations that involve the use of expiratory muscles.

#### Statistical Analysis

Data were analyzed using SPSS Statistics version 24.0 (IBM Software, Chicago, Illinois, US, 2018). Baseline characteristics were compared using Kruskal-Wallis test, Pearson Chi-Square test or Fishers exact test. Raw data are presented as median (Q1–Q3) or *n* (%). Statistical significance was accepted when *p* < 0.05.

CPAP strategies were compared over time using linear mixed-effect regression models, accounting for the relation between multiple measurements of the same lamb with a first-order autoregressive covariance structure on residuals. Group, time and group^*^time interaction were included in the model as fixed factors and random effects. To ensure that the estimated value of the parameters remained between 0–100% and 21–100%, logit transformations were used on the SaO_2_ (Ln(X/(1-X))) and FiO_2_ (Ln(79/100^*^(X-0.21)/(1–79/100^*^(X-0.21)))). The data included in the analysis were obtained either throughout the study duration, including during iPPV, or until reaching the ethical endpoint of intubation. Group *p*-values were used to compare means between groups. *Post-hoc* analyzes were performed using the False Discovery Rate. Data are presented as the median (Q1–Q3) of the group effect in the first 30 min after birth.

As rescue interventions were allowed, a comparison between groups did not reflect the effect of CPAP alone. To clarify the impact of iPPV and additional caffeine treatments on the results, the effect of successful interventions was explored using Wilcoxon Signed-Rank Test. Interventions were deemed successful if lambs continued spontaneously breathing on CPAP after iPPV or if breathing improved after the additional caffeine administration.

JVP was compared between CPAP levels using a Kruskal-Wallis Test. UTP and CPAP pressures were compared with a Wilcoxon Signed-Rank Test. The effect of a change in CPAP pressure was analyzed using Friedman's test as missing values were completed using the mean value of the group.

## Results

Lambs allocated to the LCPAP (*n* = 6), HCPAP (*n* = 6), or dynamic HCPAP (*n* = 7) groups showed no differences in baseline characteristics at birth (Table 1). In the dynamic HCPAP group, CPAP was reduced after a median (Q1–Q3) of 13.4 (8.4–20.3) min after birth, when PBF was 339 (288–423) mL/min and SaO_2_ was 91 (86–96) %, while supported by a FiO_2_ of 0.59 (0.29–0.68).

**Table 1:** Baseline characteristics.

	**LCPAP (*n* = 6)**	**HCPAP (*n* = 6)**	**Dynamic HCPAP (*n* = 7)**	***p-*value**
Birth weight (kg)	3.2 (2.7–3.8)	3.5 (3.0–3.7)	3.4 (3.0–3.8)	0.908
Twins (*n*, %)	6/6 (100%)	4/5 (80%)^*^	7/7 (100%)	0.278
Gender (male *n*, %)	2/6 (33%)	3/6 (50%)	2/7 (29%)	0.844
Meconium (*n*, %)	2/6 (33%)	2/5 (40%)^*^	2/7 (29%)	1.000
pH at birth	7.31 (7.19–7.35)	7.31 (7.27–7.34)	7.26 (7.24–7.27)	0.124
PaCO_2_ at birth (mmHg)	50.4 (43.6–69.3)	55.3 (51.6–58.1)	49.7 (47.4–55.9)	0.467
PaO_2_ at birth (mmHg)	11.0 (5.5–16.0)	14.7 (11.1–18.2)	9.7 (6.5–17.3)	0.594
SaO_2_ at birth (%)	20.7 (4.4–31.4)	27.2 (20.4–42.6)	8.7 (6.6–43.4)	0.310

*Data presented as n (%) or median (Q1–Q3)*.

* *n = 1 missing value*.

### Part I: Comparing CPAP Strategies

**Figure 2:**
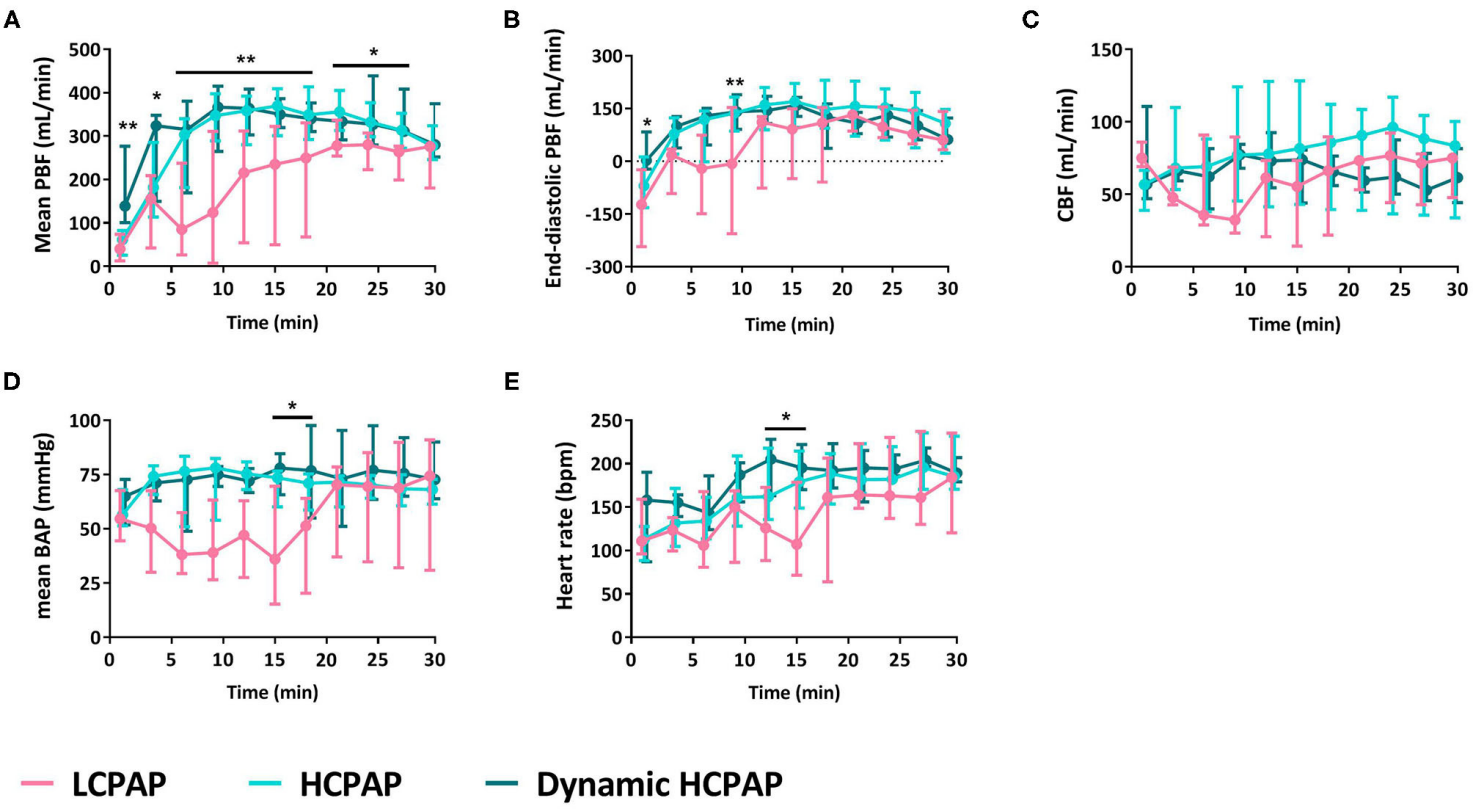
Cardiovascular outcomes. Median (Q1–Q3) of mean **(A)** and end-diastolic **(B)** pulmonary blood flow (PBF), cerebral blood flow (CBF, **C**), mean brachial artery pressure (BAP, **D**), and heart rate **(E)** in lambs receiving LCPAP vs. HCPAP vs. dynamic HCPAP in the first 30 min after birth (t0 = cord clamping). All parameters were compared over time using linear mixed-effect regression models. One (*) or two asterisks (**) indicate significant difference between the LCPAP vs. (dynamic) HCPAP or LCPAP vs. both HCPAP groups, respectively.

During the first 30 min after birth, mean (*p* = 0.002) and end-diastolic (*p* = 0.016) PBF [median (Q1–Q3) over 30 min] were significantly lower in the LCPAP group compared to the HCPAP and dynamic HCPAP groups (Figures 2A,B and Table 2). There was no difference in CBF (*p* = 0.166) or CBF variability (*p* = 0.218) between the LCPAP, HCPAP and dynamic HCPAP groups over the 30 min period after birth (Figure 2C and Table 2). BAP (*p* = 0.037) was lower in the LCPAP group compared to the HCPAP and dynamic HCPAP group (Figure 2D and Table 2). Heart rates were lower (*p* = 0.028) in the LCPAP group compared to the dynamic HCPAP group but were not different to the HCPAP group (Figure 2E and Table 2).

**Table 2:** Physiological parameters during the 30 min experimental period.

	**LCPAP (*n* = 6)**	**HCPAP (*n* = 6)**	**Dynamic HCPAP (*n* = 7)**	***p*-value**
Mean PBF (mL/min)	202 (48–277)	315 (221–365)	327 (269–376)	0.002
End-diastolic PBF (mL/min)	47 (−82 to 134)	127 (13–176)	114 (63–152)	0.016
CBF (mL/min)	65 (37–78)	73 (41–106)	66 (52–81)	0.166
CBF variability (%)	38 (17–62)	17 (14–22)	18 (15–23)	0.218
BAP (mmHg)	52 (34–68)	71 (62–77)	73 (63–84)	0.037
Heart rate (beats/min)	134 (101–174)	173 (139–205)	188 (161–207)	0.028
Breathing rate (breaths/min)	14 (0–50)	73 (38–85)	51 (31–61)	0.009
Inter-breath variability (%)	53 (32–77)	32 (23–55)	38 (29–54)	0.412
Rescue Intervention, *n* (%)	5/6 (83%)	1/6 (17%)	5/7 (71%)	0.082
Intubation rate, *n* (%)	3/6 (50%)	0/6 (0%)	0/7 (0%)	0.041
Pneumothorax rate, *n* (%)	1/6 (17%)	0/6 (0%)	0/7 (0%)	0.632
FiO_2_	0.69 (0.32–0.96)	0.42 (0.31–0.68)	0.59 (0.29–0.84)	0.286
AaDO_2_ (mmHg)	307 (122–490)	125 (74–200)	148 (75–344)	0.241
SaO_2_ (%)	48 (11–92)	88 (61–93)	88 (57–94)	0.273
PaCO_2_ (mmHg)	90.0 (81.0–131.0)	76.0 (65.0–101.5)	109.0 (56.8–146.8)	0.303
pH	6.87 (6.72–6.69)	7.08 (6.91–7.18)	6.87 (6.70–7.16)	0.097
Glucose (mmol/L)	2.7 (1.7–3.3)	1.5 (1.1–2.3)	1.6 (1.3–2.5)	0.435
Lactate (mmol/L)	8.2 (6.3–10.7)	4.2 (2.6–6.4)	5.3 (3.1–8.4)	0.050
Base excess (mmol/L)	−19.3 (−23.8 to −14.2)	−8.7 (−12.2 to −5.2)	−13.3 (−19.7 to −6.6)	0.044

*Data presented as n (%) or median (Q1–Q3)*.

**Figure 3:**
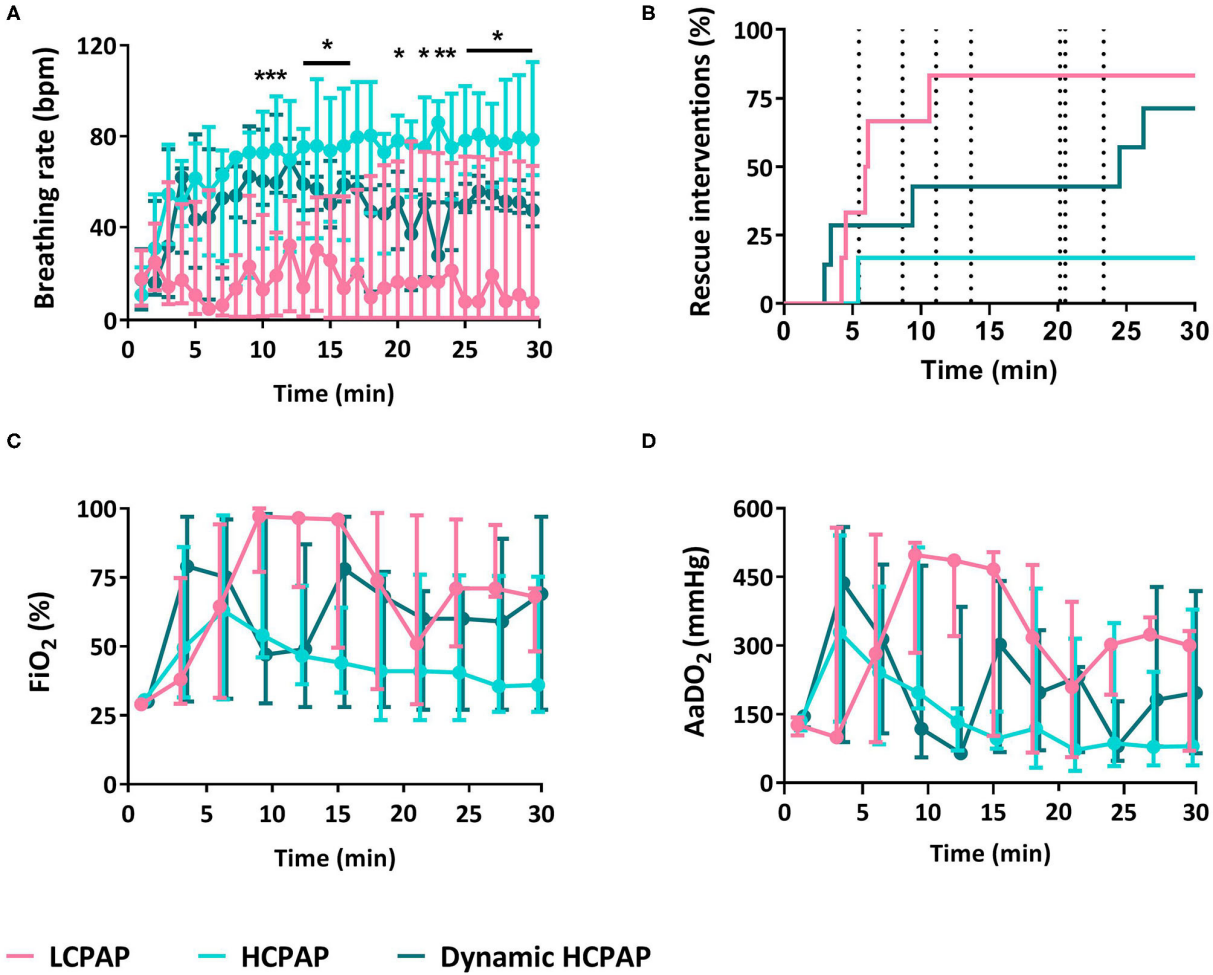
Breathing and support. Median (Q1–Q3) breathing rate **(A)**, rescue interventions **(B)**, fraction of inspired oxygen (FiO_2_, **C**), and alveolar to arterial differences in PO_2_ (AaDO_2_, **D**) in lambs receiving LCPAP vs. HCPAP vs. dynamic HCPAP in the first 30 min after birth (time 0 = cord clamping). All parameters were compared over time using linear mixed-effect regression models. One (*) or two asterisks (**) indicate significant difference between the LCPAP vs. (dynamic) HCPAP or LCPAP vs. both HCPAP groups, respectively. Dotted vertical lines **(B)** indicates the point of CPAP change in the dynamic HCPAP group.

Breathing rates were lower in the LCPAP group (*p* = 0.009) compared to the HCPAP and dynamic HCPAP groups, whereas inter-breath variability was not different between groups (*p* = 0.412, Figure 3A and Table 2). The need for rescue interventions was statistically similar (*p* = 0.082) between groups, although only 1/6 (17%) HCPAP treated lambs received rescue treatments, whereas 5/6 (83%) LCPAP, and 5/7 (71%) dynamic HCPAP lambs received rescue interventions (Figure 3B and Table 2). Nevertheless, more LCPAP lambs [3/6 (50%)] reached the point at which intubation was required, compared with [0/6 (0%)] HCPAP and [0/7 (0%)] dynamic HCPAP lambs (*p* = 0.041). During post-mortem examination, 1/6 (17%), 0/6 (0%), and 0/7 (0%) lambs of the LCPAP, HCPAP, and dynamic HCPAP group, respectively, had developed a pneumothorax (*p* = 0.632).

**Figure 4:**
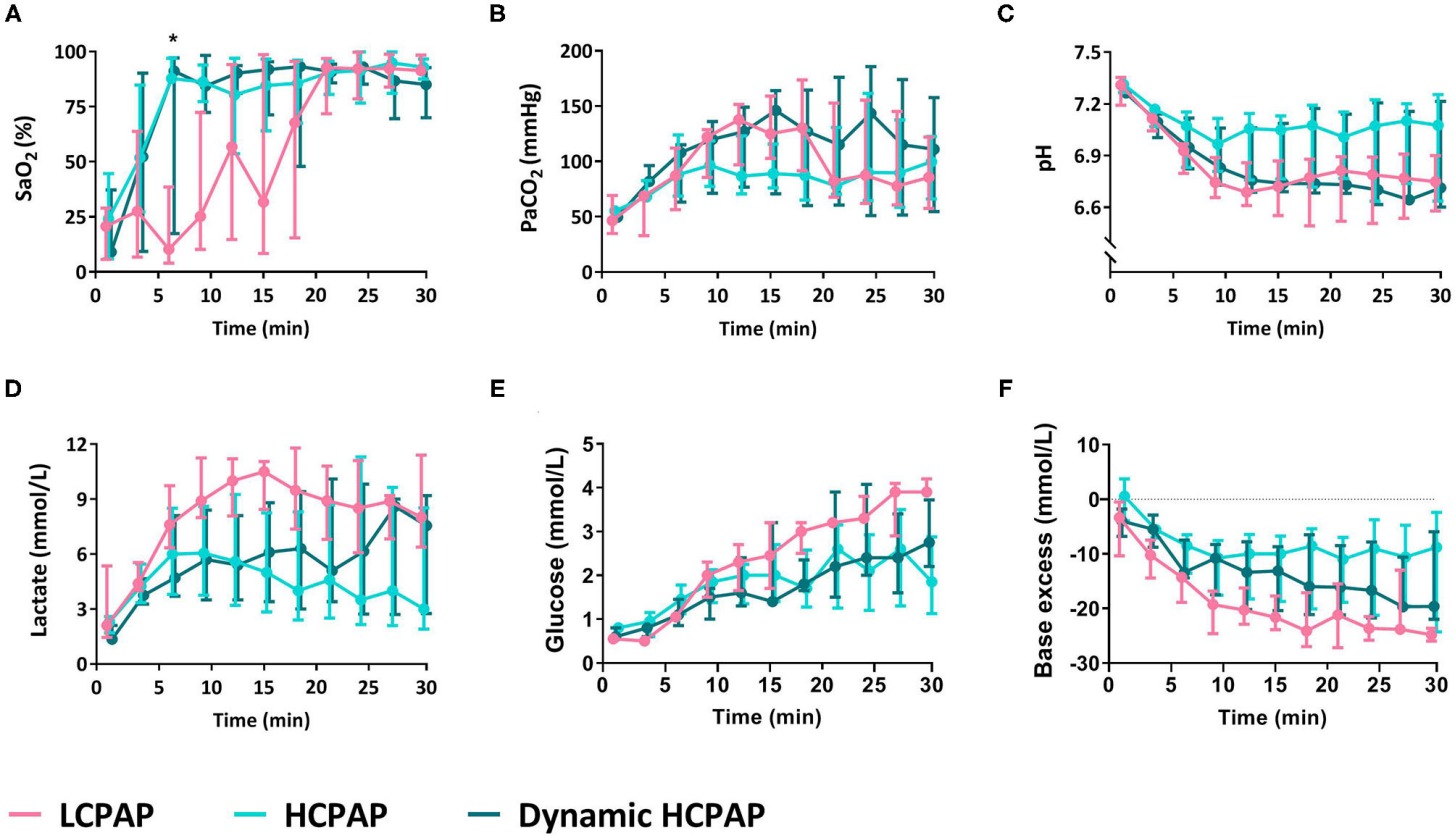
Blood gas parameters. Median (Q1–Q3) percentage of oxygen saturation of arterial blood (SaO_2_, **A**), partial pressure of carbon dioxide in arterial blood (PaCO_2_, **B**), pH **(C)**, lactate **(D)**, glucose **(E)**, and base excess **(F)** in lambs receiving LCPAP vs. HCPAP vs. dynamic HCPAP in the first 30 min after birth (time 0 = cord clamping). All parameters were compared over time using linear mixed-effect regression models. One asterisk (*) indicates the significant difference between the LCPAP vs. HCPAP group.

There were no differences in FiO_2_ (*p* = 0.286), AaDO_2_ (*p* = 0.241), SaO_2_ (*p* = 0.273), PaCO_2_ (*p* = 0.303), pH (*p* = 0.097), and glucose (*p* = 0.435) between groups (Figure 4 and Table 2). Lactate levels tended (*p* = 0.050) to be greater in the LCPAP group and base excess was lower (*p* = 0.044) in the LCPAP group, compared to the HCPAP group (Figures 3C,D, 4 and Table 2).

**Figure 5:**
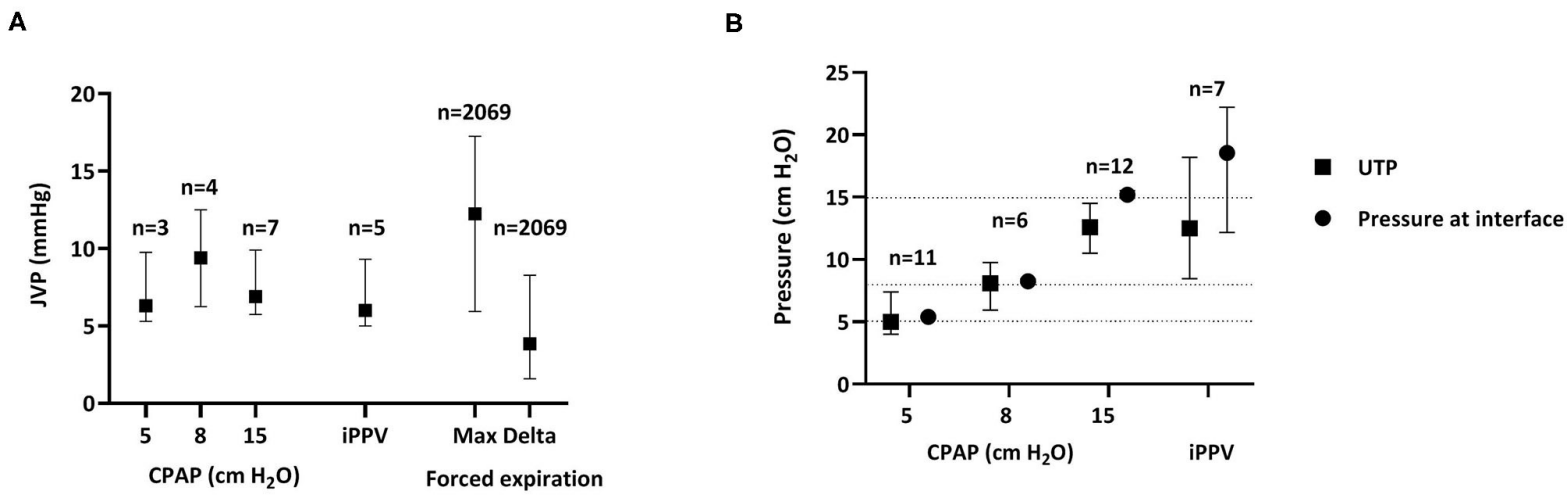
Jugular venous pressure and upper tracheal pressure. **(A)** Jugular venous pressures [JVP; median (Q1–Q3)] measured during the first 30 min after birth at different CPAP levels and during iPPV as well as the maximum (Max) pressure achieved and the change in pressure (Delta) associated with body movements or forced expirations; *n* = the number of animals during CPAP or iPPV, whereas *n* = the number of breaths with active forced expiration. JVPs were compared between groups using Kruskal-Wallis Tests. **(B)** Pressures [median (Q1–Q3)] measured simultaneously in the upper trachea (UTP, filled squares) and CPAP interface (nasal prongs, filled circles) during CPAP and iPPV throughout the entire experiment (30 + 15 min) *n* = animal number. Pressures were compared using Wilcoxon Signed-Rank Tests. Dotted horizontal line indicates 5, 8, and 15 cmH_2_O CPAP.

Baseline JVP levels were measured for each CPAP level over the first 30 min after birth, to avoid pressure changes associated with movement and breathing. Baseline JVP levels were similar at CPAP levels of 5 cmH_2_O [8.0 (5.1–12.4)], 8 cmH_2_O [9.4 (5.3–13.4)], and 15 cmH_2_O [8.6 (6.9–10.5)] and were also similar during iPPV 5.4 [(4.9–9.4) mmHg, *p* = 0.571]. However, JVP levels were increased, by an average of 3.8 (1.6–8.3) mmHg, when lambs were actively exhaling or utilizing expiratory braking maneuvers (indicated by airway pressures above CPAP). These activities resulted in maximum JVPs of 12.3 (5.9–17.3) mmHg (Figure 5A).

Baseline UTP levels were measured for each CPAP level throughout both phases of the experiment (0–45 min) to avoid pressure changes associated with movement and breathing. When a CPAP of 5 cmH_2_O was given, pressures measured at the CPAP interface (nasal prongs) were similar to those measured in the upper trachea, below the larynx [CPAP vs. UTP, 5 cmH_2_O: 5.4 (5.1–5.6) vs. 5.0 (4.0–7.4), *p* = 0.953]. Similarly, at a CPAP of 8 cmH_2_O the pressures measure at the CPAP interface [8.3 (8.1–8.5) cmH_2_O] were similar to UTPs [8.1 (6.0–9.8) cmH_2_O; *p* = 0.715]. However, when 15 cmH_2_O of CPAP was applied, UTP was lower than the pressure applied at the CPAP interface [12.6 (10.5–14.5) vs. 15.2 (14.9–15.5) cmH_2_O, respectively, *p* = 0.013]. Similarly, during iPPV, mean airway pressures measured in the UT were significantly less than that measured at the CPAP interface [12.5 (8.5–18.2) vs. 18.6 (12.2–22.2), respectively, *p* = 0.028; Figure 5].

#### Rescue Interventions

In the first 30 min after birth, 11 lambs received 9 additional dosages of caffeine and/or 13 periods of iPPV via the nasal prongs. Lambs were ventilated with an inflation rate of 50 (30–50) bpm and inter-breath variability of 11 (8–40) % for a duration of 3:08 (2:27–9:22) min (Table 3).

**Table 3:** Successful rescue interventions.

**Time**	**Caffeine (*****n*** **=** **4)**			**iPPV (*****n*** **=** **10)**		
	**Before**	**1 min after**	***p*-value**	**Before**	**Immediately after**	***p*-value**
Breathing rate (bpm)	9 (3–15)	27 (18–49)	0.043	13 (7–22)	34 (25–53)	0.005
Inter-breath variability (%)	89 (68–109)	88 (52–101)	0.893	97 (57–122)	40 (31–76)	0.037
PBF (mL/min)	197 (61–264)	290 (143–319)	0.043	115 (91–183)	287 (234–436)	0.015
CBF (mL/min)	68 (40–86)	63 (38–86)	0.345	38 (26–69)	77 (51–102)	0.009
BAP (mmHg)	79 (47–96)	71 (48–76)	0.225	46 (40–56)	71 (66–81)	0.005
Heart rate (bpm)	135 (107–163)	159 (139–201)	0.345	104 (81–123)	165 (137–208)	0.009
pH				6.724 (6.631–6.806)	6.631 (6.587–6.706)	0.115
PaCO_2_ (mmHg)				137 (126–162)	149 (120–161)	0.575
SaO_2_ (%)				12 (3–32)	90 (44–99)	0.005
AaDO_2_ (mmHg)				497 (454–544)	197 (154–445)	0.017

*Data is presented as median (Q1–Q3). Interventions were deemed successful if breathing improved after caffeine administration (4/9) or if lambs continued on CPAP after iPPV (10/13)*.

On 5/9 (56%) occasions, caffeine improved breathing and increased mean PBF. On the 10/13 (77%) occasions that iPPV was deemed successful, the first inflation was transmitted to the lungs after 4 (2–29) s and increased breathing rate, mean PBF, heart rate, SaO_2_, and AaDO_2_ (Table 3). In 3/13 (23%) animals, ventilation did not reach the lungs and intubation was required.

### Part II: Changing CPAP Levels

**Figure 6:**
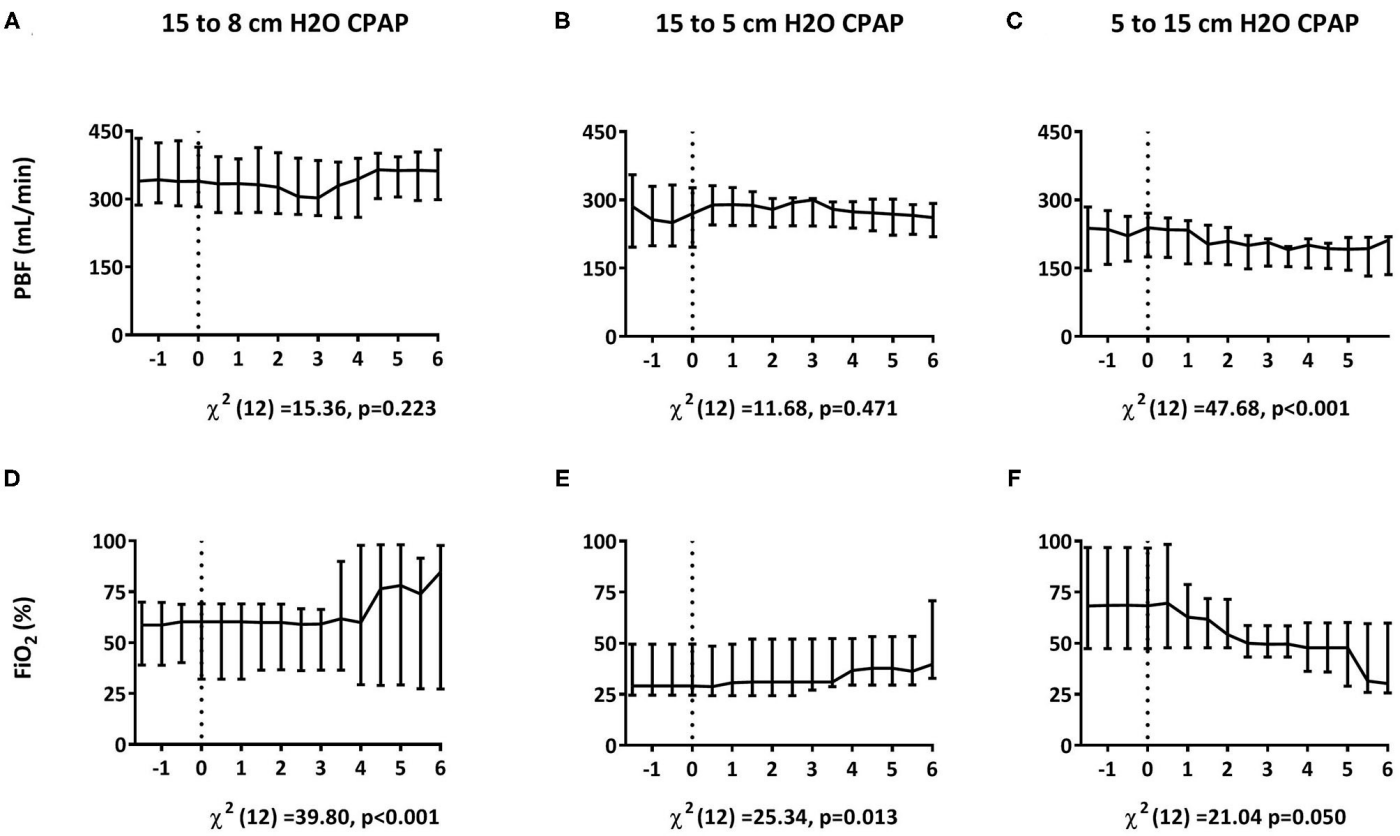
Effect of changing CPAP levels. Changes in pulmonary blood flow [PBF; **A–C**; median (Q1–Q3)] and fraction of inspired oxygen [FiO_2_; **D–F**; median (Q1–Q3)] measured in response to changes in CPAP levels from 15 to 8 cmH_2_O within 30 min after birth **(A,D)** and from 15 to 5 cmH_2_O **(B,E)** or 5 to 15 cmH_2_O **(C,F)** at 30 min after birth. Parameters were compared over time using Friedman's tests. *t* = 0 indicates the start of CPAP change.

Reducing CPAP from 15 to 8 or from 15 to 5 cmH_2_O, increased the FiO_2_ requirement, without affecting mean PBF, CBF or any of the blood gas parameters (Figure 6 and Table 4). Increasing CPAP from 5 to 15 cmH_2_O, reduced the FiO_2_ requirement and resulted in a small (~10%) but statistically significant decrease in mean PBF that took ~5 min to manifest (Figure 6). Otherwise, increasing CPAP levels had no effect on CBF, breathing rate, PaCO_2_, and SaO_2_ (Table 4).

**Table 4:** Effect of changing CPAP levels.

	**15–8 cmH_**2**_O (*n* = 9)**	**15–5 cmH_**2**_O (*n* = 5)**	**5–15 cmH_**2**_O (*n* = 7)**
CBF (mL/min)	66 (51–90) to 71 (67–77) mL/min, $X_{\left(12\right)}^{2}$ = 0.74, *p* = 0.805	86 (38–91) to 71 (37–101) mL/min, $X_{\left(12\right)}^{2}$ = 4.88, *p* = 0.962	59 (46–89) to 55 (51–69) mL/min, $X_{\left(12\right)}^{2}$ = 7.01, *p* = 0.857
Breathing rate (bpm)	66 (35–91) to 60 (52–64) bpm, $X_{\left(12\right)}^{2}$ = 14.14, *p* = 0.292	74 (67–127) to 70 (63–109) bpm, $X_{\left(12\right)}^{2}$ = 17.99, *p* = 0.116	48 (36–88) to 80 (79–81) bpm, $X_{\left(12\right)}^{2}$ = 7.91, *p* = 0.792
PaCO_2_ (mmHg)	133.0 (79.4–169.3) to 149.1 (80.4–186.3) mmHg, $X_{\left(2\right)}^{2}$ = 3.68, *p* = 0.159	60.8 (56.8–94.9) to 60.2 (45.9–81.5) mmHg, $X_{\left(2\right)}^{2}$ = 4.50, *p* = 0.105	81.4 (68.0–97.5) to 76.1 (63.0–96.0) mmHg, $X_{\left(2\right)}^{2}$ = 1.00, *p* = 0.607
SaO_2_ (%)	92 (87–95) to 86 (53–94) %, $X_{\left(2\right)}^{2}$ = 1.00, *p* = 0.607	91 (81–93) to 85 (83–96) %, $X_{\left(2\right)}^{2}$ = 1.50, *p* = 0.472	88 (78–90) to 85 (59–96) %, $X_{\left(2\right)}^{2}$ = 4.00, *p* = 0.135

*Data is presented as median (Q1–Q3) before change vs. 6 min after change*.

## Discussion

This is the first study to investigate the effect of high-CPAP, applied non-invasively, on cardiorespiratory function at birth in spontaneously breathing animals. Contrary to our hypothesis, high-CPAP did not impede the normal increase in PBF at birth, nor reduce CBF or increase JVP in spontaneously breathing preterm sheep immediately after birth. Instead, high-CPAP resulted in higher PBF, heart rate and BAP compared to low-CPAP, while showing no effect on CBF, CBF variability or the incidence of pneumothoraces. The increased mean and end-diastolic PBF also indicates that HCPAP lambs sooner achieved a lower PVR compared to the LCPAP lambs, that was substantially lower than the systemic venous resistance. While CPAP levels had little impact on JVP, large fluctuations in JVP were observed in all groups and were associated with active expirations that involved the use of expiratory muscles (mostly abdominal). In addition, lambs receiving high-CPAP had a higher breathing rates, tended to receive fewer rescue interventions and required significantly fewer intubations. Overall, high-CPAP was more successful in supporting preterm lambs throughout the neonatal transition at birth. After stabilization, reducing the CPAP level from 15 to 8 cmH_2_O resulted in a higher FiO_2_ requirement, while increasing the CPAP level from 5 to 15 cmH_2_O reduced the FiO_2_ requirement, but also caused a small reduction in PBF. Thus, we found no evidence to indicate that the use of high-CPAP immediately after birth causes pulmonary overexpansion (as indicated by a reduction in PBF) or compromises the cardiovascular system. As high-CPAP did not affect CBF, its stability or JVP, compared to low-CPAP, we found no evidence that suggests that high-CPAP may increase the risk of IVH.

**Figure 7:**
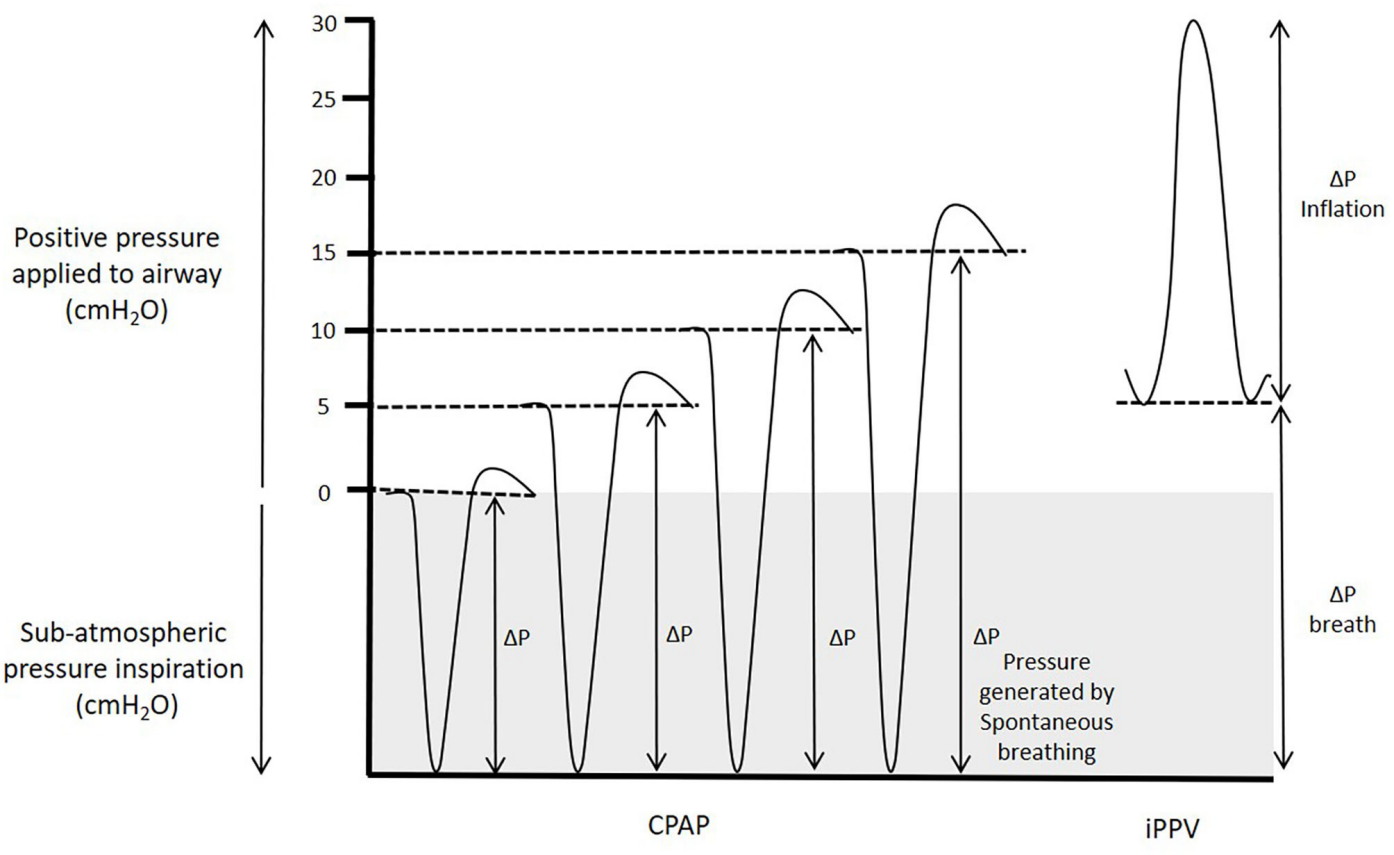
Pressure generated during CPAP and iPPV. Transpulmonary pressures (ΔP) are the primary factor that drives lung aeration after birth. During spontaneous breathing, thoracic expansion generates sub-atmospheric intrathoracic pressures, which when combined with CPAP increase the effective transpulmonary pressure applied to the lung. While intrathoracic pressures must transiently increase above the CPAP level to effect expiration, the CPAP level is effectively the highest external pressure applied to the airways and the mean intrathoracic airway pressure is considerably lower than the CPAP level. In contrast, during iPPV, PEEP is the lower limit of the applied pressure to the airways and the transpulmonary pressure that drives airway liquid clearance results from pressure increases above PEEP resulting in a mean airway pressure considerably higher than the PEEP.

Our results are in marked contrast with previous findings showing that end-expiratory pressures above 8 cmH_2_O cause pulmonary overexpansion and reduce PBF in intubated and mechanically ventilated animals (38–41). Increasing PEEP during iPPV is thought to decrease venous return to the right atrium and increase PVR by increasing alveolar pressures above alveolar capillary pressures, causing capillary closure and a reduction in PBF (39). While these studies were the best available evidence for predicting how high-pressure support affected cardiovascular function at birth, the different PBF responses between studies highlights that the two respiratory support modes are distinctly different. The primary differences being that; (i) superimposing sub-atmospheric intrathoracic pressures (due to spontaneous breathing) on a background of high-CPAP, produces different intrathoracic pressure gradients than intubation and iPPV with PEEP (Figure 7); and (ii) intubation and mechanical ventilation removes the role of the larynx, whereas CPAP and spontaneous breathing includes it.

The differential effects of spontaneous breathing and iPPV on PBF are well-established in adult physiology and the ability of individual breaths to increase PBF has been shown directly in fetal sheep (49) and indirectly in newborn infants (50). When infants breathe spontaneously during CPAP, the intrathoracic pressure phasically decreases below the CPAP level. As such, the CPAP level is effectively the highest external pressure applied to the airways, although pressures in the lower airways must briefly increase above the CPAP level to effect expiration. Nevertheless, the mean airway pressure is lower than the CPAP level measured at the mouth opening. However, during iPPV with PEEP, the PEEP level is the lower limit of the externally applied pressure and pressures phasically increase above this with each inflation (Figure 7). As such, the mean airway pressure is higher than the PEEP level and is substantially higher than occurs with a similar CPAP level during spontaneous breathing. Indeed, we found that during iPPV, lambs had a mean airway pressure of 18.6 cmH_2_O, despite using a PEEP of 5 cmH_2_O.

As adduction of the larynx can seal the airways and prevent air from enter or leaving the lungs (6–8), it can regulate the pressure in the lower airways and protect the lungs from high pressures and hyperinflation. It also allows pressurization of the airways and thorax during active expiration, as occurs during a Valsalva maneuver, for example. This is consistent with the concept that preterm newborns commonly use expiratory breaking maneuvers, either generated by post-inspiratory diaphragmatic contractions and/or laryngeal closure to help defend end-expiratory gas volumes during expiration (22, 51). We found that, when giving 15 cmH_2_O of CPAP or iPPV, only a proportion (~75%) of the pressure applied at the nasal prongs was transmitted through the larynx into the upper trachea. In contrast, at lower CPAP levels (at 5 and 8 cmH_2_O), all of the pressure applied at the nasal prongs was transmitted through the larynx into the upper trachea. Thus, it is possible that the larynx was closing to seal the airways near the end of expiration during high-CPAP, but remained open throughout the respiratory cycle at lower CPAP pressures. This may explain why studies in intubated preterm lambs observed higher pneumothorax rates when using PEEP ≤ 15 cmH2O (38, 41), while a recent study (37) found that the use of similar pressures applied non-invasively during iPPV in preterm infants did not increase the risk of adverse events.

In both the fetus and newborn, apnea or unstable breathing (varying in depth and rate), causes the larynx to close and only open during a breath (5–8). In contrast, during regular stable breathing, the larynx remains mostly open, but can briefly close to effect expiratory braking. However, lambs supported with 15 cmH_2_O were breathing continuously in a regular stable pattern and, as such we would expect the larynx to be mostly open. Nevertheless, glottic closure during expiration could be part of complicated breathing maneuvers induced by high-CPAP levels. Indeed, we noted that active expirations were common in these lambs, which likely reflects a physiological response to the increase in pressure that may also have included expiratory braking maneuvers during the expiratory phase of the breathing cycle (51).

It is also pertinent to note that in this study, the high-CPAP was applied from birth, when the lungs are initially liquid filled. In most previous studies, the lungs were aerated before the effect of high-PEEP levels on PBF was examined. This is consistent with our finding that increasing CPAP levels from 5 to 15 cmH_2_O at 30 min after birth caused a small (~10%) decrease in PBF, although this was substantially less than the decrease (~40%) observed when PEEP was increased from 4 to 12 cmH_2_O during iPPV at 20 min after birth (39). Nevertheless, the timing for when high-CPAP levels are applied to the airways may influence whether or not high pressures adversely affect PBF. Indeed, a sustained inflation (to 35 cmH_2_O) for up to 1 min does not adversely affect PBF when applied during lung aeration (52). Why this should occur is unclear, but it is possible that the initial stimulus for the increase in PBF overwhelms the adverse effect of increased alveolar pressure (52). It has recently been shown that a neural reflex, activated in response to liquid leaving the airways and entering lung tissue, triggers a global increase in PBF, which overrides all other influences such as oxygenation (19, 53). Thus, both the type and timing of application to the airways appear to determine how high airway pressures influence the cardiovascular system at birth.

At birth, pulmonary gas exchange is dependent on the available surface area (i.e., how much of the lung is aerated) and the partial pressure gradient for the respiratory gases, which for oxygen is largely determined by the FiO_2_. When the surface area is small, a large partial pressure gradient for oxygen is required for adequate oxygen exchange, whereas with increasing lung aeration, the surface area increases and so the required partial pressure gradient decreases. This finding is consistent with previous studies that have shown high-PEEP levels increase lung aeration and oxygenation and lowers the oxygen requirement (38, 39, 42, 43, 54). While we found no statistically significant differences between groups in FiO_2_, SaO_2_, or AaDO_2_, there was a clear trend toward a higher AaDO_2_ and FiO_2_ requirement and lower SaO_2_ in the low-CPAP group. The absence of a significant difference likely results from the large variability associated with application of rescue therapies (iPPV and additional caffeine) and differences in physical stimulation levels, which are difficult to standardize. Nevertheless, the close relation between CPAP level and FiO_2_ requirement was clearly indicated by changing CPAP levels. To maintain a similar SaO_2_, decreasing CPAP levels increased the FiO_2_ requirement, whereas increasing CPAP levels reduced the FiO_2_ requirement. This is consistent with the concept that higher CPAP levels increases the available area for gas exchange. Whilst supplemental oxygen stimulates spontaneous breathing and improves oxygenation, as too much oxygen can cause harm (through hyperoxia) it is important to limit the use of high O_2_ concentrations (10, 55, 56). Our results, and those of our previous study (30), indicate that this can be achieved through the use of high-CPAP, which increases lung aeration, improves gas exchange, and reduces the FiO_2_ requirement. Future studies are required to confirm the role of high-CPAP on improving lung aeration, and to investigate the interaction between CPAP level and FiO_2_, to find the optimal support strategy that can be tested in preterm infants at birth.

Contrary to our hypothesis, the dynamic high-CPAP strategy was not superior to high-CPAP. Indeed, our findings indicate that the dynamic high-CPAP group may have benefitted by remaining at the high CPAP level for longer and delaying the decrease. Whilst these lambs continued to have a high PBF and SaO_2_ following the reduction in CPAP, they needed a higher FiO_2_ to maintain their oxygenation levels and most probably their level of breathing activity. As preterm infants often only require a maximum of 8 cmH_2_O CPAP in the neonatal ward, it is necessary to decrease the CPAP level at some stage, but the questions of when and upon what indication, remains unknown. Clearly, the strategy of dynamic high-CPAP strategy needs further investigation, to identify the desirable moment for reducing the CPAP level.

Our model of spontaneously breathing preterm animals over time introduces some bias into the study because it incorporates strategies to stimulate lambs to breath. As a result, differential use of rescue interventions between groups effected the results by improving study outcomes in groups with higher rates of rescue interventions. As the interventions were required more frequently in the low-CPAP and dynamic high-CPAP group, mostly after reducing the CPAP level, the differences between these groups and the high-CPAP group were potentially reduced. The early drop-out of animals in the low-CPAP group, which occurred after they had reached the ethical endpoint where intubation was required, likely further reduced the difference between the groups. As such, high-CPAP levels likely benefitted these preterm sheep to a greater degree than we were able to demonstrate.

It is unclear why lambs in all groups experienced high pCO_2_ and low pH levels, although it is likely to be mostly metabolic in origin as indicated by the high lactate and large negative base excess levels. Indeed, at 15–30 min, lambs in the HCPAP group had a high SaO_2_ (>90%) while receiving a relatively low FiO_2_ (~30%), but still had high PCO_2_ levels. As the solubility and exchange potential for CO_2_ is almost 30 times greater than O_2_, a problem with CO_2_ exchange in the absence of a problem with O_2_ exchange is unlikely and so the high PCO_2_ is most likely secondary to the metabolic acidosis. As lambs were not hypoxic, the metabolic acidosis is unlikely to have resulted from anaerobic glucose metabolism. It could have resulted from activation of the sympathetic nervous system due to birth related stress or to a cold stimulus as the lambs were not sedated. Indeed, while we measured core body temperatures and applied external heat as required, activation of non-shivering thermogenesis may have contributed to maintaining core body temperature. Furthermore, as caffeine is a non-specific phosphodiesterase inhibitor (57, 58), it may have enhanced these cAMP mediated responses. This is consistent with the finding that HCPAP lambs received less caffeine and tended to have higher pH and lower PCO_2_ levels. While acidosis and high PCO_2_ levels can adversely affect the cardiovascular system and reduce PBF, we found no evidence for this as all lambs, that completed the 30 min experimental period, achieved high PBFs with significant left-to-right shunting despite many of them had a low pH; the latter is indicative of a low pulmonary vascular resistance.

In summary, high-CPAP levels resulted in PBF levels that were markedly higher than those seen with low-CPAP levels when applied from birth, and successfully supported preterm lambs throughout the neonatal transition after birth. We did not find any indication that high-CPAP caused pulmonary overexpansion, compromised the cardiovascular system or increased risk factors for IVH when given directly at birth. However, at 30 min after birth, while increasing CPAP levels reduced the FiO_2_ requirement, it also caused a small reduction in PBF. This indicates that the timing after birth, and the type of positive pressure respiratory support applied, determines whether high airway pressures adversely affect the cardiovascular system at birth.

## Data Availability Statement

The datasets supporting the conclusions of this article will be made available by the authors upon request to the corresponding author.

## Ethics Statement

Study procedures were performed in accordance with the National Health and Medical Research Council of Australia guidelines for care and use of experimental animals and were approved by Monash University MMCA Ethics committee. All research staff exposed to the sheep were vaccinated against Q fever (Q-Vax CSL, Australia).

## Author Contributions

TM, KC, KR, AP, and SH made substantial contributions to conception and design of the study. TM, KC, KR, JD, AD, AM, VZ, GP, CR, and SH performed the experiments and obtained the data. TM, SH, and AP were responsible for data analysis and interpretation and drafted the first version of the manuscript. All authors provided feedback and approved the final version of the manuscript. All authors included in this paper fulfill the criteria of authorship.

## Conflict of Interest

The authors declare that the research was conducted in the absence of any commercial or financial relationships that could be construed as a potential conflict of interest.

## Abbreviations

AaDO_2_: alveolar to arterial differences in PO_2_
BAP: Brachial Artery Pressure
CBF: Carotid Blood Flow
CPAP: Continuous Positive Airway Pressure
FiO_2_: Fraction of inspired Oxygen
HCPAP: High-CPAP
iPPV: intermittent Positive Pressure Ventilation
JVP: Jugular Vein Pressure
LCPAP: Low-CPAP
UTP: Upper Tracheal Pressure
PaCO_2_: Partial Pressure of Carbon Dioxide in arterial blood
PBF: pulmonary blood flow
PEEP: Positive End-Expiratory Pressure
SaO_2_: percentage of oxygen saturation of arterial blood.
